# The Neuroprotection of KIBRA in Promoting Neuron Survival and Against Amyloid β-Induced Apoptosis

**DOI:** 10.3389/fncel.2019.00137

**Published:** 2019-04-12

**Authors:** Lin Song, Shi Tang, Lingling Dong, Xiaolei Han, Lin Cong, Jixin Dong, Xiaojuan Han, Qinghua Zhang, Yongxiang Wang, Yifeng Du

**Affiliations:** ^1^Department of Neurology, Shandong Provincial Hospital affiliated to Shandong University, Jinan, China; ^2^Department of Neurology, Dongying People’s Hospital, Dongying, China; ^3^Fred and Pamela Buffett Cancer Center, University of Nebraska Medical Center, Omaha, NE, United States; ^4^Eppley Institute for Research in Cancer and Allied Diseases, University of Nebraska Medical Center, Omaha, NE, United States

**Keywords:** KIBRA, Alzheimer’s disease, amyloid-β, apoptosis, neuroprotection

## Abstract

**Background**: Recent research has identified the nucleotide polymorphisms of KIdney and BRAin expressed protein (KIBRA) to be associated with cognitive performance, suggesting its vital role in Alzheimer’s disease (AD); however, the underlying molecular mechanism of KIBRA in AD remains obscure.

**Methods**: The AD animal model (APP/PS1 transgenic mice) and KIBRA knockout (KIBRA KO) mice were used to investigate pathophysiological changes of KIBRA *in vivo*. Mouse hippocampal cell line (HT22) was used to explore its molecular mechanism through KIBRA CRISPR/Cas9-sgRNA system and KIBRA overexpression lentivirus *in vitro*.

**Results**: Aged APP/PS1 mice displayed increased neuronal apoptosis in the hippocampus, as did KIBRA KO mice. KIBRA deficiency was closely related to neuronal loss in the brain. In addition, knockdown of KIBRA in neuronal cell lines suppressed its growth and elevated apoptosis-associated protein levels under the stress of Aβ_1–42_ oligomers. On the contrary, overexpression of KIBRA significantly promoted cell proliferation and reduced its apoptosis. Moreover, through screening several survival-related signaling pathways, we found that KIBRA inhibited apoptosis by activating the Akt pathway other than ERK or PKC pathways, which was further confirmed by Akt-specific inhibitor MK2206.

**Conclusion**: Our data indicate that KIBRA may function as a neuroprotective gene in promoting neuron survival and inhibiting Aβ-induced neuronal apoptosis.

## Introduction

Alzheimer’s disease (AD) is the most common neurodegenerative disease characterized by progressive cognitive deterioration, with the number of people affected by AD being projected to double over the next 35 years (Prince et al., 2015). The extracellular accumulation of amyloid-β (Aβ) plaques and the intracellular formation of neurofibrillary tangles (NFTs) in the brain are known to play critical roles in the pathogenesis of AD (Jack et al., 2010). According to the “Amyloid Cascade Hypothesis” (Hardy and Higgins, 1992; Hardy and Selkoe, 2002), Aβ deposition is the initial pathological trigger in the disease, which subsequently leads to the formation of NFTs, neuronal cell death, and ultimately the expression of dementia (Reitz, 2012). Evidence from genetic, neuropathological, and cell biological research strongly suggests that therapeutic interventions targeting Aβ may be beneficial for patients with AD (Selkoe, 2013; Ising et al., 2015); however, attempts to identify therapeutic approaches by targeting Aβ have not been successful so far (Doody et al., 2014; Salloway et al., 2014). A potential reason may be that AD is a polygene disease resulting from complex interactions between heredity and environment over the lifespan (Van Duijn et al., 1994). Thus, the urgent need for understanding the Aβ production and clearance process promotes investigators looking for new protective genes against AD.

KIdney and BRAin expressed protein (KIBRA) is a member of the WWC family of proteins (Kremerskothen et al., 2003). The genome-wide single (GWS) nucleotide polymorphism (SNP) analysis has recently shown that the genetic locus encoding KIBRA is significantly associated with human memory performance (Papassotiropoulos et al., 2006; Nacmias et al., 2008; Schaper et al., 2008). In mammals, KIBRA is highly expressed in kidney and memory-related brain regions, such as the hippocampus and cortex (Kremerskothen et al., 2003). Thus, KIBRA is also known as the memory-related gene (Johannsen et al., 2008). Since memory impairment is a cardinal clinical feature of AD, different reports have recently established the close association of KIBRA with AD (Almeida et al., 2008; Burgess et al., 2011). The first study on KIBRA and AD revealed that the T allele of KIBRA rs17070145 was significantly associated with increased risk for very-late-onset AD (Rodriguez-Rodriguez et al., 2009). On the contrary, a subsequent study showed that the T allele non-carriers had an increased risk of late-onset AD in two cohorts (Corneveaux et al., 2010).

Animal studies also provide evidence for the association between KIBRA and memory performance (Yoshihama et al., 2009; Wang et al., 2014). KIBRA functions as a novel bidirectional regulator of synaptic and structural plasticity in hippocampal neurons, and formation of long-term memory, which is highly relevant to the cognitive processes and their pathologies (Blanque et al., 2015; Heitz et al., 2016). More recently, research showed that training rats in the T maze task significantly increased the accuracy of rats’ performance as well as increased the expression of KIBRA in the prefrontal cortex (Wang et al., 2014). Additionally, KIBRA may affect higher brain function by regulating α-amino-3-hydroxyl-5-methyl-4-isoxazole-propionate receptor (AMPAR) trafficking and synaptic plasticity. Adult KIBRA knockout mice showed reduced long-term potentiation and depression as well as deficits in contextual fear learning and memory (Makuch et al., 2011).

Recent research indicated that KIBRA was required for autophagy to function properly (Jin et al., 2015). The absence of KIBRA caused defects in the formation of autophagic vesicles and autophagic degradation. Impairment in the function of autophagic vesicles in the brain will lead to the abnormal clearance of misfolded proteins, eventually resulting in cell apoptosis. Therefore, changes in KIBRA expression levels might affect cell apoptosis. The associations between the KIBRA and AD have been verified (Hayashi et al., 2010; Vyas et al., 2014; Zhang et al., 2014; Liu et al., 2015; Jin et al., 2016; Witte et al., 2016), but the underlying molecular mechanism of KIBRA in AD remains obscure. Given the pivotal role of Aβ-induced neuronal apoptosis in the pathogenesis of AD, we hypothesize that KIBRA may be involved in Aβ-induced neuronal apoptosis.

In this experimental study, our investigations discovered an interesting phenomenon that KIBRA knockout (KIBRA KO) mice displayed increased neuronal apoptosis in the hippocampus. It is emerging evidence that the absence of KIBRA leads to neuronal loss. In addition, overexpression of KIBRA in neuronal cell lines significantly promoted its proliferation and inhibited Aβ-induced apoptosis through activation of Akt pathway but not ERK or PKC pathways, which was further confirmed by Akt-specific inhibitor MK2206. In summary, our data demonstrate that KIBRA functions as a neural protective gene that promotes cell survival and inhibits Aβ-induced apoptosis. Thus, KIBRA has been regarded as a potentially therapeutic target for AD treatment.

## Materials and Methods

### Animals

The APPswe/PSEN1dD9 (APP/PS1) mice were purchased from Model Animal Research Center of Nanjing University. WWc1^tm1.1Rlh^ mice were purchased from the Jackson Laboratory (No. 024415; Bar Harbor, ME, USA). KIBRA was completely knocked out in all tissues of this strain. The mice were bred in the Animal Facility of Shandong Provincial Hospital affiliated to Shandong University, and were housed with a 12/12 h light/dark cycle and with *ad libitum* access to food and water. All mice were humanely killed with an overdose of anesthetics and then perfused transcardially with saline for biochemical analysis and with 4% paraformaldehyde for histological analysis. All experimental procedures were approved by the Animal Care and Use Committee of Shandong Provincial Hospital affiliated to Shandong University and were conducted following the institutional guidelines.

### Aβ_1–42_ Oligomers Preparation

Amyloid β-Protein_(1–42)_ was purchased from BACHEM (Switzerland, CH) and prepared according to a slightly modified method (Bellacosa et al., 1991). Briefly, Aβ_1–42_ peptide was suspended in hexafluoroisopropanol (Sigma Aldrich, St. Louis, MO, USA) to reach 1 mM and stored as a dried film at −80°C. The solution was placed in a biological safety cabinet for volatilization of HFIP to form a peptide film, which was re-suspended in dimethyl sulfoxide (DMSO; Sigma-Aldrich) and then sonicated in a water bath for 10 min. Phosphate-buffered saline (PBS) containing 0.05% sodium dodecyl sulfate was used to re-suspend the peptide, and then further diluted with cold PBS to 11.1 nM prior to incubation for 2 weeks at 4°C. The oligomeric form of Aβ_1–42_ was verified by an electron microscope. Before treatment of cells, the oligomer solution was centrifuged at 13,000 rpm for 10 min at 4°C.

### Cell Culture and Treatment

Mouse hippocampal neuronal cell line (HT22) was obtained from Jennio Biological Technology (Guangzhou, China). Cells were cultured in Dulbecco’s modified Eagle’s medium (DMEM; Hyclone/Thermo Fisher Scientific, USA) supplemented with 10% fetal bovine serum (FBS; Gibco, Argentina), 100 U/ml penicillin and 100 μg/ml streptomycin at 37°C in humidified atmosphere containing 5% CO_2_. Before the treatments, the medium was replaced by serum-free DMEM medium. Cells were treated with various doses of Aβ_1–42_ oligomers (0, 1, 2, 3, 4, and 5 μM). To analyze the protective effect of KIBRA, the KIBRA CRISPR and overexpression cells were treated with 1 μM Aβ_1–42_ oligomers for 24 h.

### KIBRA CRISPR and Overexpression Cell Models

To generate KIBRA-knockdown cells using CRISPR-Cas9 gene editing system, Cas9 vectors and sgRNA targeting the KIBRA gene were cloned into GV371-CMV-hSpCas9-SV40-Puro and GV371-U6-KIBRA sgRNA-SV40-EGFP, respectively. The LV-Cas9 and LV-sgRNA lentiviruses were produced by GeneChem (Shanghai, China). We constructed three sgRNA oligonucleotides and tested the effects of the three sgRNAs on gene silencing prior to the experiments. The sgRNA sequence was as follows: CATCAGTGATGAGTTACCGC. LV-Cas9 was seeded in HT22 cells with a multiplicity of infection (MOI) of 50 after 5–7 days of puromycin selection at a final concentration of 3 μg/ml; LV-sgRNA was seeded, and the empty vector was used as controls. After 5–7 days, the KIBRA knockdown cells and their control cells were harvested, respectively.

Lentivirus (GV367) particles carrying Wwc1 and the control vectors were constructed by GeneChem (Shanghai, China). The specific primers of mouse Wwc1 were: (forward) 5′-ACAGTGATGAATCGGAAG-3′ and (reverse) 3′-GTAGTGACAGAGCAGAGA-5′. The lentiviral constructs were transfected into HT22 cells supplemented with 5 μg/ml polybrene (Sigma, USA). The medium was changed 24 h after infection, and Green Fluorescent Protein (GFP)-positive cells were sorted after 72 h. The upregulated efficiency was tested by western blot.

### Immunofluorescence

Freshly frozen Brain tissue sections were fixed with 4% paraformaldehyde for 15 min. Afterward, the brain slices were blocked in 5% normal goat serum for 1 h at 37°C followed by the incubation of primary antibodies 4°C overnight. The staining was visualized by labeling the corresponding secondary antibodies and then counter-stained with DAPI (4′,6′-diamidino-2-phenylindole) for 10 min at 37°C. The following antibodies used: mouse anti-beta III Tubulin (1:1,000; Abcam), Alexa Fluor 594 conjugated goat anti-mouse IgG (1:300; Proteintech). All images were collected and analyzed with an LSM780 confocal laser scanning microscope combined with the upright microscope.

### Immunohistochemistry

Brain tissues were fixed in 4% paraformaldehyde and embedded in paraffin blocks. After deparaffinization and rehydration, brain slices were boiled in citric buffer (10 mM Citric acid, pH 6.0) for 4 min for antigen retrieval. The slides were incubated with 3% hydrogen peroxide for 30 min to block endogenous peroxidase activity. Subsequently, sections were incubated overnight at 4°C with the following primary antibodies: rabbit anti-β-Amyloid (1:400; Cell Signal Technology), mouse anti-Tau (1:1,000; Cell Signal Technology) and rabbit anti-P-Tau Thr181 (1:1,000; Cell Signal Technology). Following removal of the antibodies *via* several rinses with PBS, the respective antibodies were detected using the avidin-biotin-peroxidase complex method. Slides were lightly counterstained with hematoxylin and then were dehydrated with sequential ethanol. All images were analyzed by optical microscopy.

### TUNEL Assay

*In situ* cell death detection kit, fluorescein (Roche, Switzerland) was used to detect and quantify apoptosis, which was based on the labeling of DNA strand breaks (TUNEL technology). Brain tissues were fixed in 4% paraformaldehyde for 15 min. Following drop-wise addition of 0.1% Triton X-100 (freshly prepared by 0.1% sodium citrate), the samples were incubated for 15 min at room temperature and washed twice with PBS for 5 min. Following drop-wise addition of 50 μl TUNEL reaction solution, the samples were incubated for 1 h at 37°C in the dark and then mounted with DAPI for nuclear counter staining. Both TUNEL and DAPI-positive cells were counted. Data are reported as TUNEL index, which was calculated by counting the total number of TUNEL-positive cells.

### Morris Water Maze Assay

Morris water maze was conducted to assess spatial learning and reference memory of the mice according to a previously described protocol (Pearson et al., 2001). Briefly, mice were trained to find a submerged escape platform in an open swimming arena. The maze was divided geographically into four equal quadrants and release points that were designed at each quadrant as N, E, S, and W. A hidden circular platform, made of Plexiglas, was located in the center of the southwest (target) quadrant, submerged 1 cm beneath the surface of the water. These were kept in fixed positions with respect to the swimming pool to allow the mouse to locate the escape platform hidden below the water surface. Each trial lasted for 60 s with an additional 30 s learning time, where mice were allowed to remain on the platform. After 6 days of learning, the mice were subjected to a probe test in which the platform was removed. Mice were analyzed for a number of times they passed previous learning time platform location (SW). Data were analyzed using Smart 3.0 software.

### Western Blot

Cells were lysed in RIPA buffer with 1% phenylmethylsulfonyl fluoride and 1% protein phosphatase inhibitor (Beyotime, China) on ice for 20 min. Proteins in the mouse tissue homogenate were extracted with RIPA buffer (Beyotime, China). Lysates were centrifuged at 12,000 rpm for 30 min at 4°C to obtain supernatants. Equal amounts of protein lysates in RIPA were separated on 8%, 10% or 12% SDS-PAGE gels before being transferred to polyvinylidene fluoride membranes (Merck Millipore, USA). The membranes were blocked with 5% milk and then incubated with primary antibodies in primary antibody dilution buffer (Beyotime, China) at 4°C overnight. The blots were probed with the following antibodies: Tau 1:1,000, P-Tau Thr181 1:1,000, Akt 1:1,000, P-Akt Ser 473 1:2,000, P-ERK1/2 Thr202/Tyr204 1:2,000, ERK1/2 1:1,000, Caspase 3 1:1,000, P-PKCζ/λ Thr410/403 1:1,000 (Cell Signal Technology, USA); KIBRA 1:500, PKCζ 1:1,000 (Santa Cruz, USA); PARP-1 1:1,000 (Abcam, UK); β-actin 1:2,000 (Proteintech, China). The membranes were subsequently incubated with peroxidase-conjugated secondary antibodies and developed using the enhanced chemiluminescence (Merck Millipore) method. Band densities were quantified by densitometry using Fluor ChemQ software.

#### Cell Viability Assay

Cell viability was assessed by the Cell Counting Kit-8 (CCK-8; Dojindo, Japan). Cells were plated on 96-well microplates at a density of 3 × 10^3^/well. Then the medium was replaced by serum-free medium before different time and concentration gradients of Aβ_1–42_ oligomers were treated. CCK-8 solution was added to each well, and the microplates were incubated for 4 h at 37°C in a 5% CO_2_ atmosphere. The absorbance was read at 450 nm.

#### Cell Proliferation Assay

Cells (2 × 10^3^/well) were dispensed with culture media (100 μl) into 96-well plates. At specified time-points (0, 24, 48, 72 and 96 h), 10 μl CCK-8 solution (CCK-8; Dojindo, Japan) was added to each well. The absorbance at 450 nm was assessed at the end of the incubation (2 h). All experiments were performed in triplicate.

#### Statistical Analysis

We used the GraphPad Prism 5.0 software (GraphPad Software Inc., La Jolla, CA, USA) for data analysis. Data were expressed as means ± SEM. Technical, as well as biological triplicates of each experiment, were performed. For continuous variables with a Gaussian distribution, differences in the means were analyzed either by one-way analysis of variance (ANOVA) with Tukey–Kramer *post hoc* tests (multiple groups) or *t*-test (two groups). For data not following a Gaussian distribution, the nonparametric Mann–Whitney test was used. Two-tailed *P* < 0.05 was considered statistically significant.

## Results

### Increase of Neuronal Apoptosis in the Brain of APP/PS1 and KIBRA KO Mice

**Figure 1:**
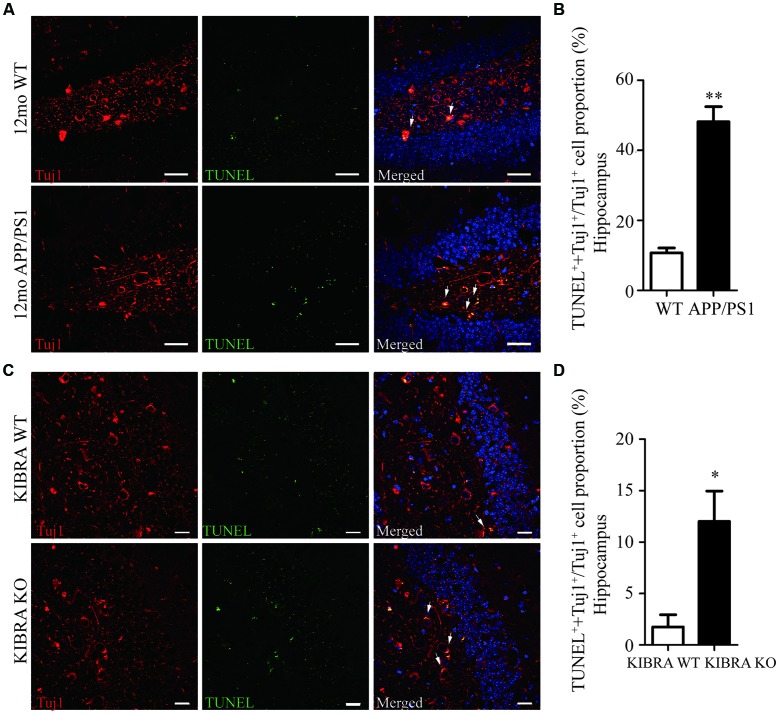
The indispensable role of KIdney and BRAin expressed protein (KIBRA) in the neuron survival. The apoptotic neurons (Tuj1, red) were analyzed by TUNEL assay (green) in APP/PS1 **(A)** and KIBRA KO **(C)** mice. Nuclei were fluorescently labeled with DAPI (blue). Compared with the wild type mice, a higher number of TUNEL positive neurons was observed in the hippocampus of APP/PS1 mice brain **(B)**. In addition, 4-month-old KIBRA KO mice brain showed significantly increased TUNEL positive neurons **(D)**, suggesting that KIBRA plays an indispensable role. Results are means ± SEM. (*n* = 5 mice in each group) from independent experiments. *p < 0.05 and **p < 0.01. Scale bar in **(A)** = 10 μm. Scale bar in **(C)** = 20 μm.

The 12-month-old APP/PS1 mice displayed pathological Aβ and tau accumulation in the brain (Supplmentary Figures S1A–G), accompany with age-dependent impairments of spatial memory and learning (Supplmentary Figures S2A–I), as expected AD mouse model. To investigate whether KIBRA exerted a protective role against neuronal apoptosis, we evaluated used by immunofluorescent staining of Tuj1 and TUNEL in APP/PS1 mice and KIBRA KO mice. Neuronal apoptotic cells in the hippocampus (Figures 1A,B) and cortex (Supplmentary Figures S3A,B) markedly elevated in aged APP/PS1 mice compared with the wild-type mice. Subsequently, KIBRA KO mice were used to confirm the function of KIBRA in neurons. The number of apoptotic neurons was significantly higher in the hippocampal area (Figures 1C,D) and cortex area (Supplmentary Figures S3C,D) of KIBRA KO mice than that in the wild-type mice, suggesting that KIBRA deficiency resulted in the neuronal apoptosis in the brain. These results provide further evidence that KIBRA plays an indispensable role in neuron survival.

### The Role of KIBRA in Promoting Cell Proliferation

To further illustrate the critical function of KIBRA *in vitro*, the mouse hippocampal neuronal cell line—HT22 was used to establish KIBRA knockdown and overexpression cell models with CRISPR/Cas9-sgRNA system and overexpression lentivirus, respectively. As indicated by GFP expression, the virus infection efficiency was up to 80% in KIBRA overexpression cells (Supplmentary Figure S4A) and 90% in the KIBRA knockdown cells (Supplmentary Figure S5A). The overexpression of KIBRA was confirmed through western blot (Supplmentary Figure S4B), and the highest KIBRA knockdown efficiency was observed in the sequence No. 01051-1 of Lenti-CRISPR/Cas9-sgRNA, which was used as the KIBRA knockdown cell model (Supplmentary Figure S5B).

**Figure 2:**
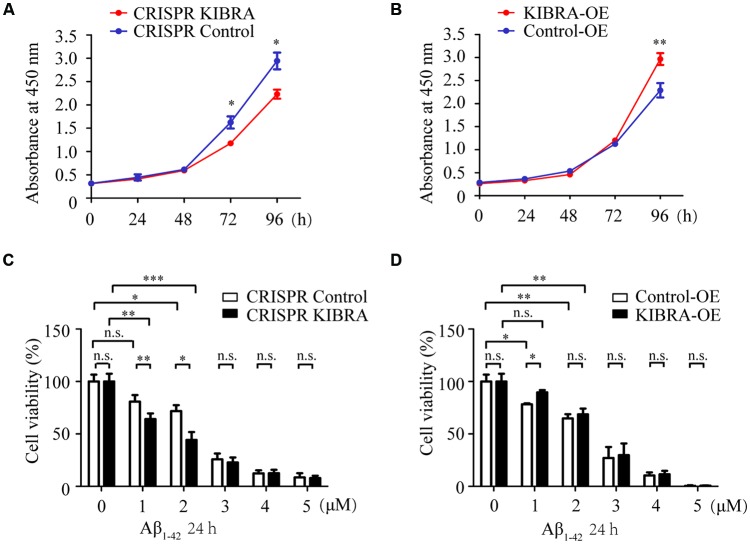
The indispensable role of KIBRA against Aβ_1–42_ oligomers-induced neurotoxicity. Cell proliferation was assessed by CCK8 assay in KIBRA cell lines. The results showed that KIBRA knockdown **(A)** significantly suppressed cell growth at 72 h and 96 h after seeding, whereas overexpression of KIBRA **(B)** started to stimulate cell proliferation at 96 h. CCK8 assay was also carried out with various doses of Aβ_1–42_ oligomers (0, 1, 2, 3, 4, and 5 μM) and Aβ_1–42_ oligomers induced the decrease of cell viability in a dose-manner **(C,D)**. Interestingly, the CRISPR KIBRA cells were more sensitive to the toxicity of Aβ_1–42_ oligomers than the CRISPR control cells **(C)**. Overexpression of KIBRA significantly promoted cell viability compared to the control group **(D)**. Results are means ± SEM (*n* = 4 per group) from independent experiments. *p < 0.05, **p < 0.01 and ***p < 0.001, n.s., not significant (*p* > 0.05).

To assess the cell proliferation of KIBRA cell models, CCK8 assay was carried at various time points (0, 24, 48, 72, and 96 h) after cells were seeded in a 96-well plate with the same original cell numbers. Interestingly, cell growth in KIBRA knockdown group gradually slowed at 72 and 96 h compared with control group, there were no significant differences among these groups at 24 and 48 h after seeding (Figure 2A). In contrast, KIBRA overexpression group showed significant elevated cell proliferation after 96 h culture but no differences were detected among these groups from 24 to 72 h (Figure 2B). Taken together, these data revealed that KIBRA was a positive regulator in cell proliferation which is consistent with the previous report (Stauffer et al., 2016).

### The Neuroprotective Effect of KIBRA Against Aβ_1–42_ Oligomers-Induced Neuron Apoptosis

Aβ-induced neuron apoptosis is believed to be a critical event in the pathogenesis of AD (Shimohama, 2000; Reitz, 2012). To assess the impact of KIBRA on Aβ-induced apoptotic cell death, CCK8 assay was carried out with various doses of Aβ_1–42_ oligomers (1, 2, 3, 4, and 5 μM) for 24 h. With the treatment of 1 and 2 μM Aβ_1–42_ oligomers, both KIBRA knockdown and control group cell viabilities apparently declined, and the former were more sensitive to neural toxicity of Aβ (Figure 2C). Moreover, the cell survival of knockdown of KIBRA under the stress of Aβ_1–42_ oligomers was shortened compared to controls. It was reasonable to believe that shortage of KIBRA made neurons more vulnerable to the toxicity of Aβ_1–42_ oligomers. Conversely, overexpression of KIBRA significantly reduced cell death compared to the control group with the treatment of 1 μM Aβ_1–42_ oligomers (Figure 2D). With the dose of Aβ_1–42_ oligomers increasing, there were no statistical significances of cell viabilities in the KIBRA cell models, mostly due to excessive toxicity. Briefly, these data strongly supported the positive role of KIBRA in promoting neuron survival and against Aβ_1–42_ oligomers-induced neurotoxicity.

**Figure 3:**
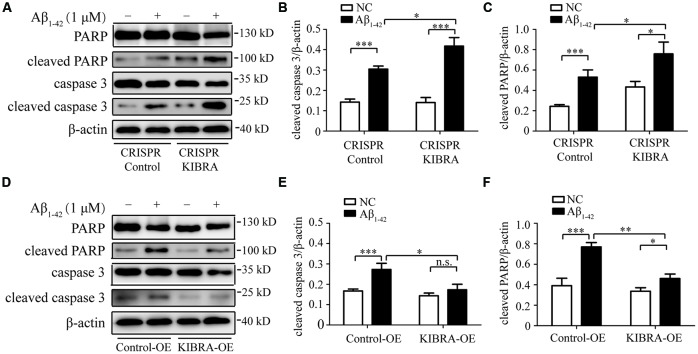
The protective function of KIBRA against Aβ_1–42_ oligomers-induced neuron apoptosis. CRISPR KIBRA cells and KIBRA overexpression cells were treated with 1 μM Aβ_1–42_ oligomers for 24 h and apoptosis-related proteins (cleaved caspase 3 and cleaved PARP) levels were measured by western blot. Aβ_1–42_ oligomers treatment significantly increased the levels of cleaved caspase 3 **(A,B)** and cleaved PARP **(A,C)** in CRISPR KIBRA cell than that of the control. There was an apparent reduction of cleaved caspase 3 **(D,E)** and cleaved PARP **(D,F)** in KIBRA overexpression cells. Results are means ± SEM (*n* = 4 per group) from independent experiments. *p < 0.05, **p < 0.01 and ***p < 0.001, n.s., not significant (*p* > 0.05).

To further examine whether Aβ_1–42_ oligomers induced neuron death in an apoptosis-associated mechanism in our study, western blot was utilized to measure several key apoptosis-related proteins, including caspase 3 and PARP. As shown in Figures 3A–C, obvious higher levels of cleaved caspase 3 and cleaved PARP were detected in CRISPR KIBRA cells than that in the control group induced by Aβ_1–42_ oligomers. Meanwhile, there was an apparent reduction of apoptosis-associated proteins in KIBRA overexpression cells compared with control cells (Figures 3D–F). These results had implications for further research to define the role of KIBRA involved in suppressing Aβ_1–42_ oligomers-induced neuron apoptosis.

### The Activation of ERK and PKC in KIBRA Cell Models After Aβ_1–42_ Oligomers Treatment

**Figure 4:**
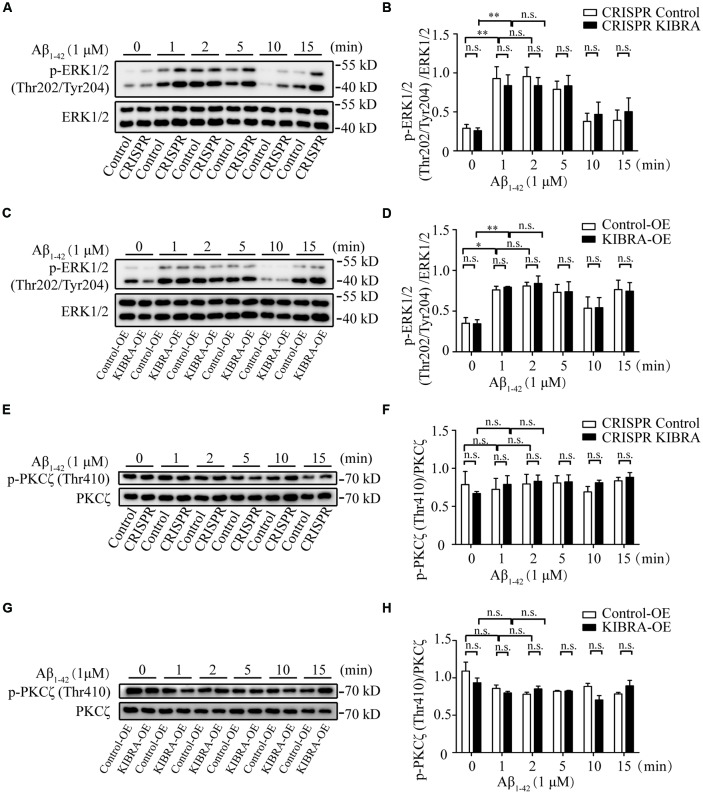
The activation of ERK and PKC pathways in KIBRA CRISPR and overexpression cells with Aβ_1–42_ oligomers treatment. KIBRA CRISPR and overexpression cell models were treated with 1 μM Aβ_1–42_ oligomers at different time points. The total ERK1/2 and phosphorylated ERK1/2 proteins were all detected by western blot **(A–D)**. As shown, the rapid activation of phosphorylated ERK (p-ERK1/2) Thr202/Tyr204 was observed at 1, 2, and 5 min after Aβ_1–42_ oligomers treatment, without changes in the levels of total ERK. Nevertheless, there were no statistical differences in the ERK activation pattern that accompany with knockdown or overexpression of KIBRA, compared to the control groups. The total PKCζ and phosphorylated PKCζ proteins were all detected by western blot **(E–H)**. The overall phosphorylated PKCζ (p-PKCζ) Thr410 remained at high levels regardless of Aβ_1–42_ oligomers treatment in KIBRA CRISPR or overexpression cells. Similarly, the activation of p-PKCζ Thr410 was not significantly different between KIBRA cell models and control groups. Results are means ± SEM (*n* = 3 per group). *p < 0.05 and **p < 0.01, n.s., not significant (*p* > 0.05).

To identify the key downstream effectors involved in KIBRA protective effect against apoptosis, we screened several survival-related signaling pathways (e.g., ERK, PKC, and Akt signaling) at time gradients (0, 1, 2, 5, 10, and 15 min) at 1 μM of Aβ_1–42_ oligomers in KIBRA cell models. As shown, the rapid activation of phosphorylated ERK (p-ERK1/2) Thr202/Tyr204 was observed at 1, 2, and 5 min after Aβ_1–42_ oligomers treatment, without changes in the levels of total ERK. Nevertheless, the differences were not statistically significant in the ERK activation pattern that accompany with knockdown or overexpression of KIBRA, compared to the control groups (Figures 4A–D). The data showed that ERK pathway did not participate in KIBRA protective effect against apoptosis.

Previous studies showed that KIBRA regulated AMPA receptor function through interaction with PKCζ (Yoshihama et al., 2009; Vogt-Eisele et al., 2014). In our study, the overall phosphorylated PKCζ (p-PKCζ) Thr410 remained at a high level regardless of Aβ_1–42_ oligomers treatment in KIBRA CRISPR or overexpression cells (Figures 4E–H). Similarly, the activation of p-PKCζ Thr410 showed no significant differences between KIBRA cell models and control cells, indicating that PKC pathway was not involved in regulating the neuroprotective role of KIBRA. These findings might be due to cell-specific regulation, or the fact that the follow-up time was too short to detect significant differences.

### The Neuroprotective Role of KIBRA in Promoting Neuron Survival Through Akt Activation

**Figure 5:**
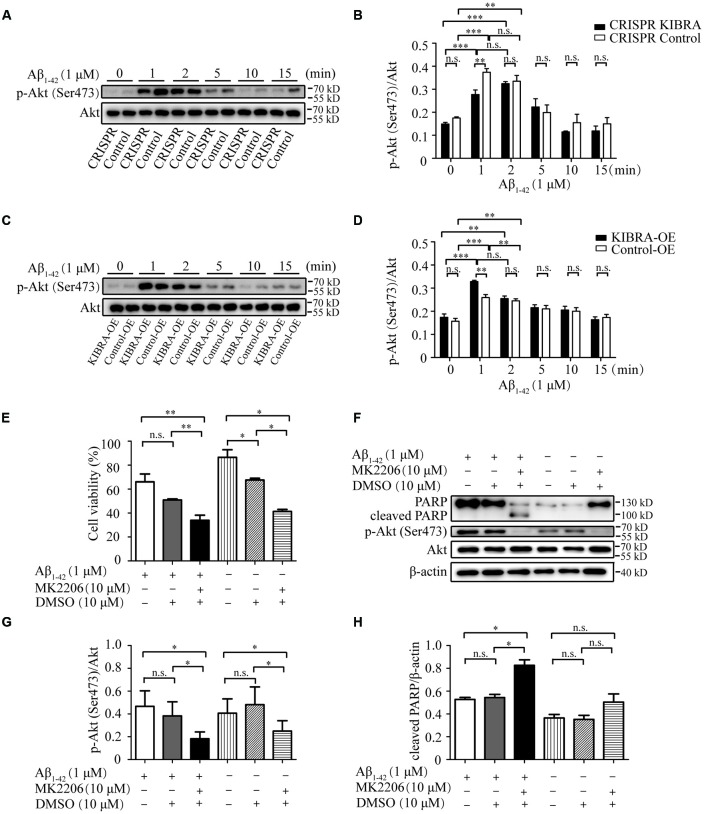
The neuroprotective role of KIBRA on promoting neuron survival through targeting Akt signaling. CRISPR KIBRA cells and KIBRA overexpression cells were treated with 1 μM Aβ_1–42_ oligomers for 0, 1, 2, 5, 10, and 15 min. The total Akt and phosphorylated Akt proteins were all detected by western blot. Western blot analysis indicated that the activation p-Akt Ser473 appeared at 1 and 2 min with Aβ_1–42_ oligomers treatment in KIBRA cell models. Nevertheless, the activation of p-Akt Ser473 at 1 min in CRISPR KIBRA was not as high as that of the control group **(A,B)**. Interestingly, although KIBRA overexpression group and control group quickly activated p-Akt Ser473 at 1 and 2 min, the level of p-Akt Ser473 was significantly elevated in KIBRA overexpression group **(C,D)**. In addition, the levels of total Akt were not affected by Aβ_1–42_ oligomers. KIBRA overexpression cells were treated with 10 μM MK2206 or 10 μM DMSO vehicle for 30 min prior to Aβ1–42 oligomers. Cell viability was measured by CCK-8 assay and the amounts of PARP, p-Akt and Akt were determined by western blot. The treatment of MK2206 significantly decreased cell viability **(E)** and inhibited p-Akt Ser473 signaling activation **(G)** induced by the treatment of Aβ1–42 oligomers. Furthermore, the prior treatment of MK2206 led to higher level of cleaved PARP compared with control group under Aβ_1–42_ oligomers treatment **(F,H)**. Results are means ± SEM (*n* = 4 per group) from independent experiments. *p < 0.05 and **p < 0.01, and ***p < 0.001, n.s., not significant (*p* > 0.05).

It was previously reported that Akt was a serine-threonine kinase activated by growth factors or survival factors through phosphatidylinositol-3-kinase (PI3K) to promote cell growth and survival (Burgering and Coffer, 1995; Datta et al., 1999; Lou et al., 2011), thus the Akt activation level was tested after the treatment with 1 μM Aβ_1–42_ oligomers at different time points. Western blot analysis indicated that the activation p-Akt Ser473 appeared at 1 and 2 min with Aβ_1–42_ oligomers treatment in KIBRA cell models. Nevertheless, the activation of p-Akt Ser473 at 1 min in CRISPR KIBRA was not as high as that of control group (Figures 5A,B). These results provided a new idea that KIBRA may regulate activation of Akt signaling under Aβ_1–42_ oligomers conditions. Interestingly, although KIBRA overexpression group and control group quickly activated p-Akt Ser473 at 1 and 2 min, the level of p-Akt Ser473 was significantly elevated in KIBRA overexpression group (Figures 5C,D). In addition, the levels of total Akt were not affected by Aβ_1–42_ oligomers. Thus, it was reasonable to believe that the function of KIBRA was mediated through the activation of Akt pathway.

To further verify the mediation of Akt in the neuroprotective function of KIBRA, KIBRA overexpression cells were treated with a specifically-allosteric Akt inhibitor—MK2206 prior to Aβ_1–42_ oligomers exposure and then the neurotoxicity was analyzed by CCK8 assay and western blot. As shown in Figure 5E, the overall neuron survival of KIBRA overexpressed cells with MK2206 treatment markedly declined compared to vehicle (DMSO) treatment group. The result showed that the presence of MK2206 significantly inhibited p-Akt Ser473 activation (Figures 5F,G). Furthermore, the prior treatment of MK2206 led to a higher level of cleaved PARP compared with the control group under Aβ_1–42_ oligomers treatment (Figures 5F,H). Given that the reduction of Akt signaling significantly attenuated the neuroprotective effect of KIBRA, KIBRA functions as a neural protective gene that promotes cell survival and inhibit Aβ-induced apoptosis.

## Discussion

Despite several studies implicating KIBRA in the progression of AD, the underlying molecular mechanism was still unknown. In this study, we provided evidence that depleting KIBRA dramatically increased neuronal apoptosis both *in vitro* and *in vivo*. Moreover, KIBRA inhibited Aβ-induced neural apoptosis by targeting key downstream effectors of Akt signaling pathway other than PKC and ERK pathway *in vitro*. In summary, KIBRA functions as a neural protective gene that promotes cell survival and inhibits Aβ-induced apoptosis.

Since KIBRA’s discovery, most studies of KIBRA have been focused on its role in learning, memory performance, and other neurodegenerative disorders, especially AD. In addition, KIBRA is involved in multiple cellular functions such as synaptogenesis, vesicle transport, transcriptional regulation, cell polarity and migration through interacting with diverse partners, which also play crucial roles in the pathogenesis of AD (Hilton et al., 2008; Xiao et al., 2011; Yoshihama et al., 2011, 2012; Ji et al., 2012). KIBRA regulates higher brain function by regulating AMPAR trafficking and synaptic plasticity through forming a tripolymer complex with protein interacting with C-kinase 1 (PICK1). Furthermore, KIBRA is also necessary for autophagy to function properly, and the absence of KIBRA caused defects in the formation of autophagic vesicles and autophagic degradation (Jin et al., 2015). Meanwhile, several studies have demonstrated the positive role of KIBRA in regulating cell proliferation, suggesting its vital role in anti-apoptosis (Stauffer et al., 2016).

Notably, Aβ accumulation and the downstream pathological events such as neuronal apoptosis play critical roles in the pathogenesis of AD. But the important problem of how KIBRA functions in AD pathogenesis is still confused. Interestingly, KIBRA KO mice showed much higher numbers of apoptotic neurons in the brain compared to control mice. Our finding provides evidence that KIBRA deficiency is closely associated with neuronal apoptosis. It will be of particular interest to explore whether the loss of KIBRA contributes to neuronal apoptosis in the AD condition. Thus, we further investigated relevant cellular mechanisms through mouse hippocampal cell line with Aβ_1–42_ oligomers treatment *in vitro*.

We investigated the effect of knockdown and overexpression of KIBRA in neuronal cells and further studied the downstream mediators. Consistent with previous reports, we observed that KIBRA knockdown significantly suppressed cell growth, whereas overexpression of KIBRA stimulated cell proliferation. Moreover, the deficiency of KIBRA showed enhanced apoptosis after treatment with Aβ_1–42_ oligomers, whereas overexpression of KIBRA rescued Aβ neurotoxicity. KIBRA not only functions as a positive regulator in cell proliferation, but also protects neurons from Aβ toxicity.

Interestingly, previous reports indicated that the activation of Akt may prevent Aβ-induced neuronal death (Yang et al., 2017). A major pathway that blocks caspase-3 activation is associated with the kinase PI3K and its downstream effector kinase, Akt. In the current study, we observed the dramatic increase of p-Akt Ser473 activation with the overexpression of KIBRA, and pretreatment with Akt specific inhibitor—MK2206 resulted in a significant increase of neuronal apoptosis. As KIBRA functions as a cytoskeletal-associated protein that provides a platform for the activation of other kinases, the distinct downstream effectors of KIBRA has been reported in the regulation of AMPA receptor trafficking, which may contribute to the differences in cell types and stimulation conditions. KIBRA KO mouse is a valuable tool to illustrate the molecular mechanism of its anti-apoptotic function, and we will make great efforts on the further exploration. In summary, our data indicated that KIBRA functions as a neuroprotective gene in promoting neuron survival and inhibiting Aβ-induced neuronal apoptosis, which is helpful for the development of a novel treatment target for AD.

## Ethics Statement

This study was carried out in accordance with the recommendations of the institutional guidelines of the Animal Care and Use Committee of Shandong Provincial Hospital affiliated to Shandong University. The protocol was approved by the Animal Care and Use Committee of Shandong Provincial Hospital affiliated to Shandong University.

## Author Contributions

LS and ST carried out the molecular studies, and drafted the manuscript. XiaoleiH and LD performed the statistical analysis. CC, JD, XiaojuanH, and QZ made substantial contributions to the animal studies. YW and YD conceived the study, obtained study funding, participated in its design and coordination and drafted the manuscript. All authors critically read and approved the final manuscript.

## Conflict of Interest Statement

The authors declare that the research was conducted in the absence of any commercial or financial relationships that could be construed as a potential conflict of interest.
