# Supplementary material for: The Neuroprotection of KIBRA in Promoting Neuron Survival and Against Amyloid β-Induced Apoptosis
Source: Front Cell Neurosci. 2019 Apr 12;13:137. doi: 10.3389/fncel.2019.00137 (PMC6473163; doi:10.3389/fncel.2019.00137)
Supplement: Supplementary file 1 [file Data_Sheet_1.docx]

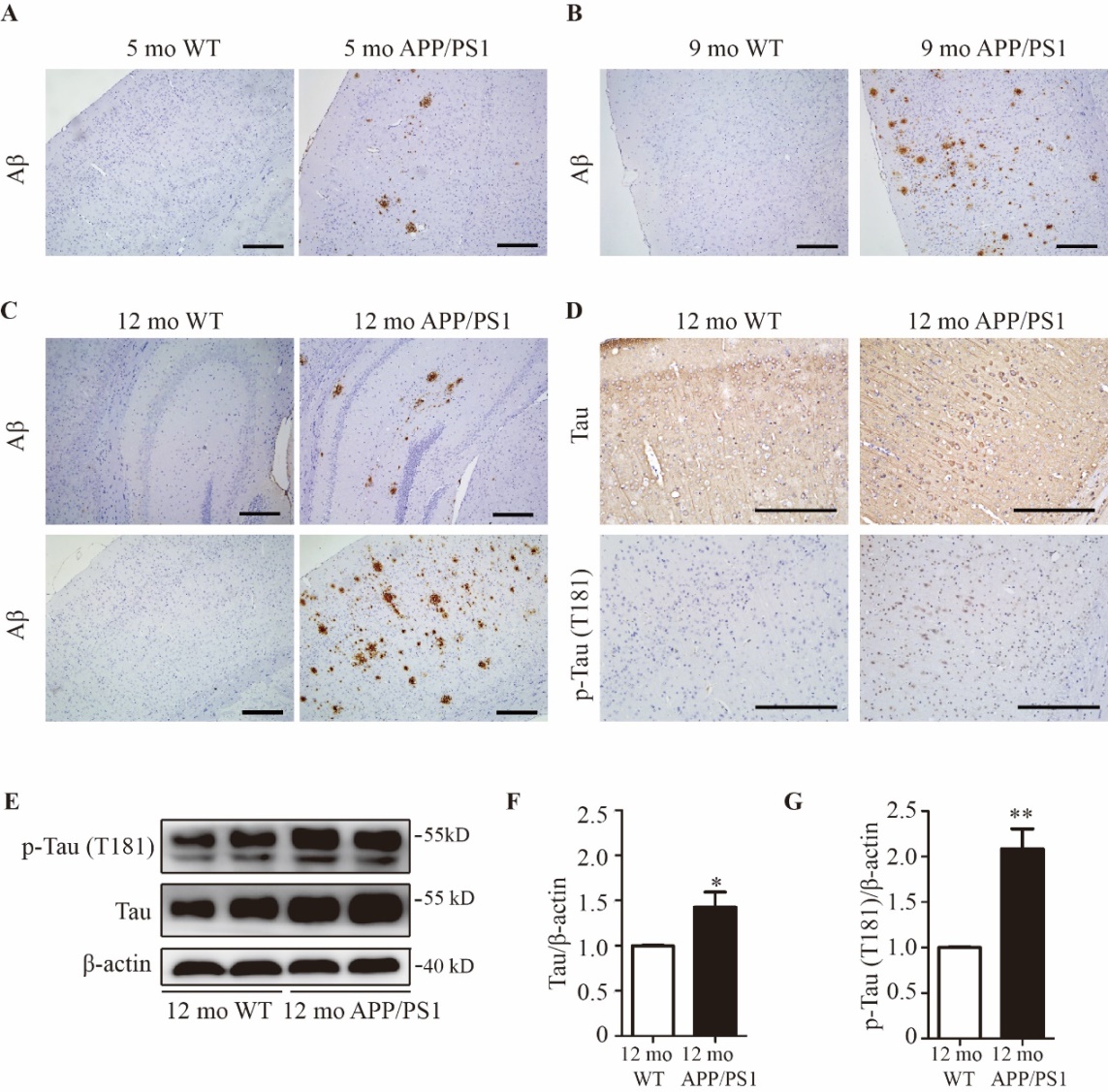
**Figure S1. Pathological changes in aged APP/PS1 mice.**

Immunohistochemical staining insets showed significant amyloid plaque burden in 5-month-old (**A**) and 9-month-old (**B**) APP/PS1 mice brain. Insets showed significantly higher amyloid plaque deposition in 12-month-old APP/PS1 mice hippocampus and cortex (**C**). Immunochemical staining showed that Tau and Tau-phosphorylation were expressed in the brain of APP/PS1 mice brain (**D**). Quantification of Tau and Tau-phosphorylation was using by western blot (**E**). The results showed that the expression of Tau and Tau-phosphorylation were consistently and significantly increased in the brain of APP/PS1 mice (**F** and **G**). Results are means ± S.E.M. (*n* = 5 mice in each group) from independent experiments. **p*<0.05 and ***p*<0.01. Scale bar for A, B and C = 20 μm. Scale bar for D= 400 μm.

**
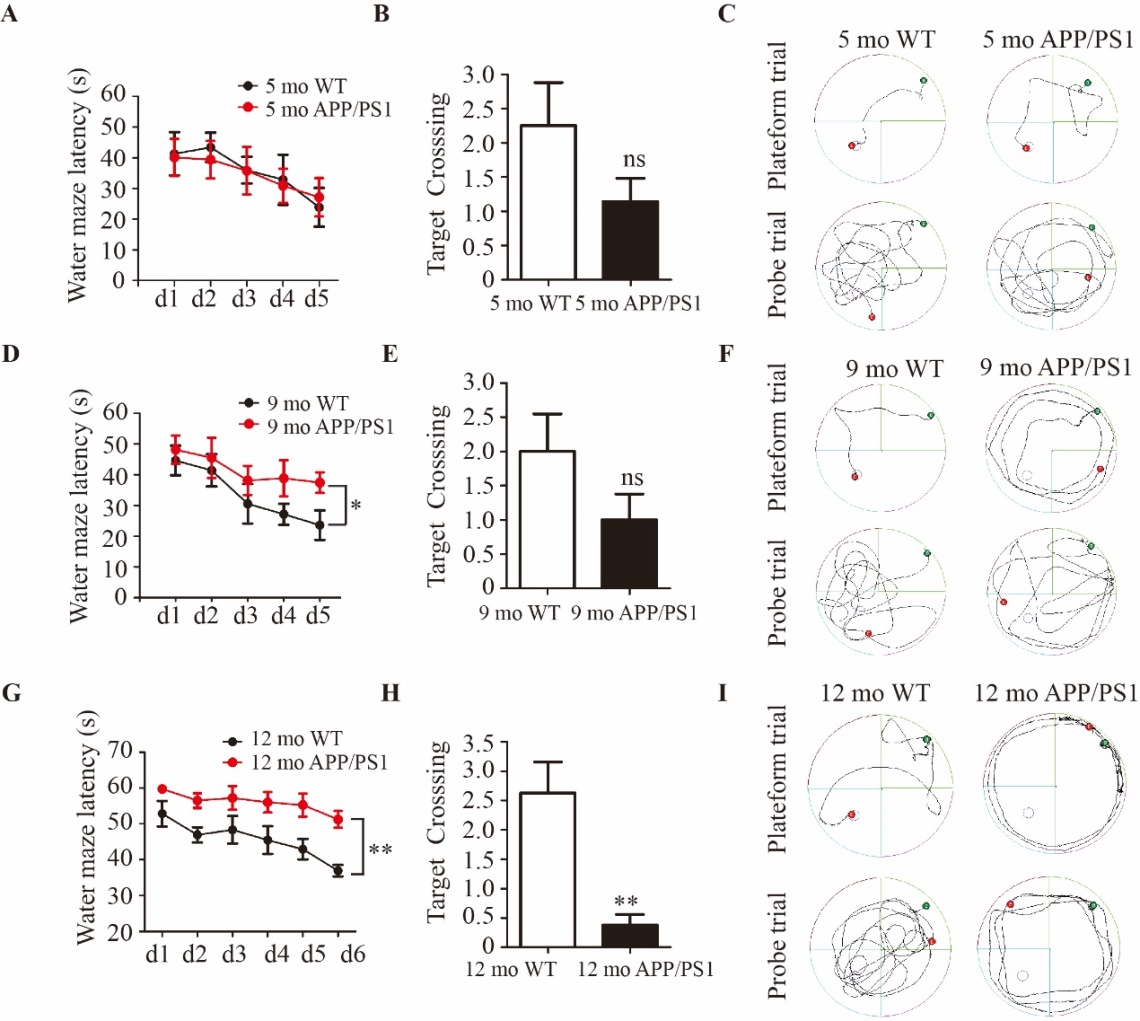
Figure S2. Age-dependent cognitive decline in aged APP/PS1 mice.**

No cognitive deficit was observed between APP/PS1 mice and control group of 5-month-old from the result of Morris water maze (**A**, **B** and **C**). By 9 months of age, the escaping latency during platform trials in APP/PS1 mice was longer than that the controls (**D** and **F**), while no obvious difference was observed in number of target crossing (**E**) in probe test. By 12 months of age, the escaping latency during platform trials in APP/PS1 mice was longer than that in wild type mice (**G**). There was a significant reduction in number of target crossing (**H**) in probe test and a longer distance traveled (**I**) during platform trials in APP/PS1 mice. Results are means ± S.E.M. (*n* = 5 mice in each group) from independent experiments. **p*<0.05 and ***p*<0.01, ns, not significant (*p* > 0.05).

**
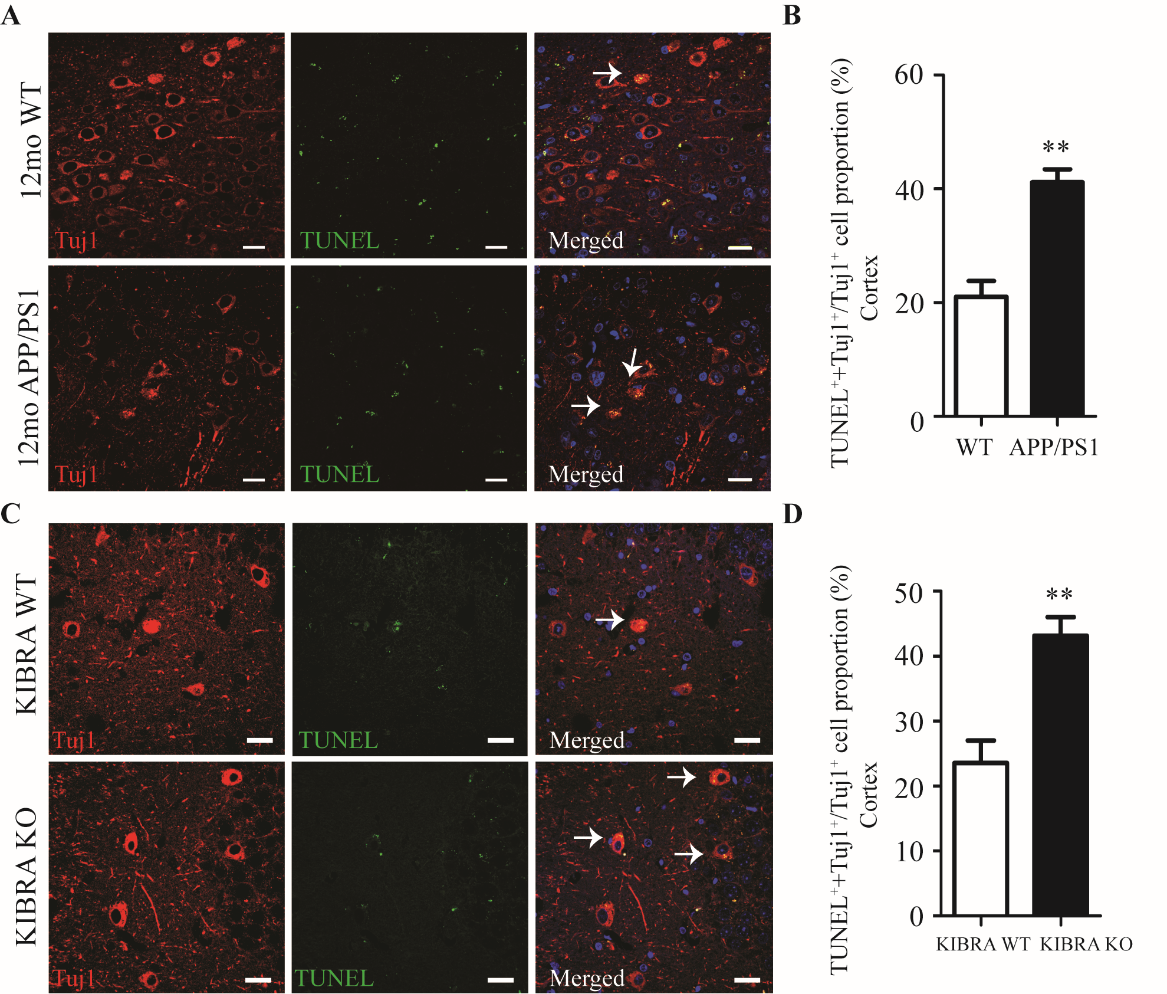
Figure S3. The indispensable role of KIBRA in the neuron survival**

The apoptotic neurons (Tuj1, red) were analyzed by TUNEL assay (green) in APP/PS1 mice (**A** and **C**). Nuclei were fluorescently labeled with DAPI (blue). Compared with the wild type mice, a higher number of TUNEL positive neurons was observed in the cortex of APP/PS1 mice (**B**). The number of apoptotic neurons was significantly higher in the cortex of KIBRA KO mice than that in the wild-type mice (**D**). Results are means ± S.E.M. (*n* = 5 mice in each group) from independent experiments. ***p*<0.01. Scale bar A and C=20 μm.


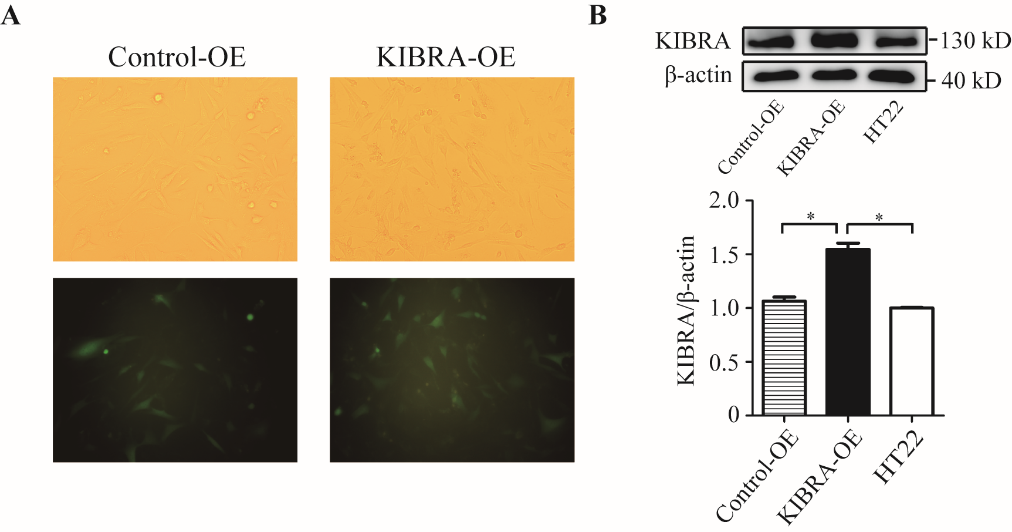
**Figure S4. Establishment of KIBRA overexpression cell models.**

The mice hippocampal neuronal cell line-HT22 cells were used to establish KIBRA overexpression cell models with KIBRA viral vector. As indicated by GFP expression, the viral infection efficiency was up to 80% (**A**). Western blot results showed that an increased level of KIBRA was significant in KIBRA overexpression cells (**B**). Results are means ± S.E.M. (n = 4 per group) from independent experiments. **p*<0.05.

**
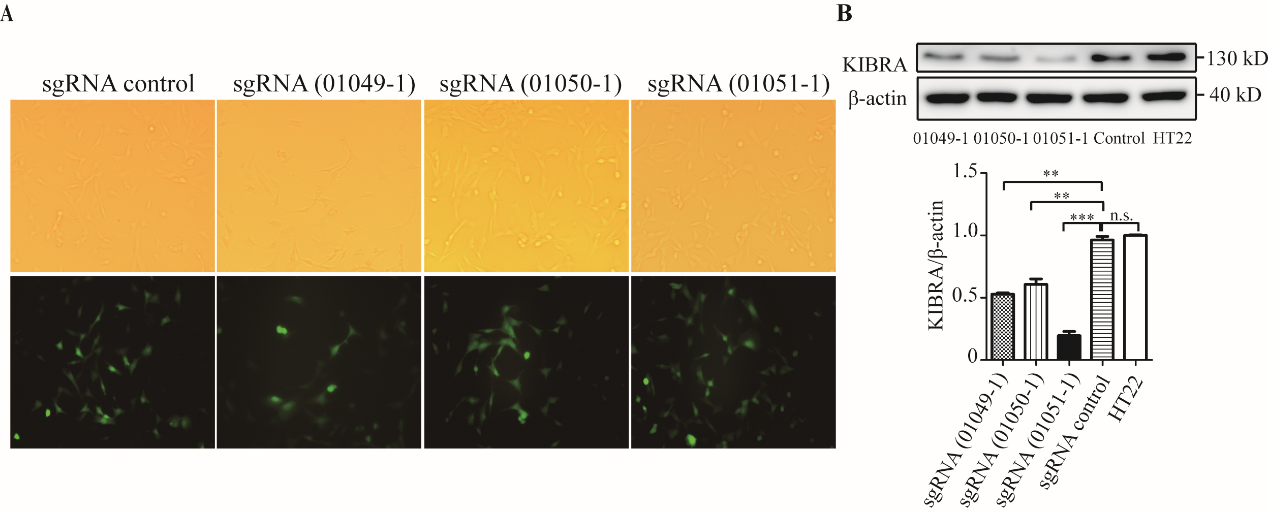
Figure S5. Establishment of KIBRA knockdown cell models.**

The mice hippocampal neuronal cell line-HT22 cells were used to establish KIBRA knockdown cell models with CRISPR/Cas9 system. As indicated by GFP expression, the viral infection efficiency was up to 90% (**A**). Western blot results showed that the highest KIBRA knockdown efficiency was observed in the Lenti-CRISPR/Cas9-sgRNA (01051-1) sequence group (**B**). Results are means ± S.E.M. (n = 4 per group) from independent experiments. ***p*<0.01 and ****p*<0.001, ns, not significant (*p* > 0.05).
